# Commentary on: Quality-of-Life Evaluation of Patients Undergoing Lumbar Discectomy Using Short Form 36

**DOI:** 10.5812/kowsar.22287523.3459

**Published:** 2012-01-01

**Authors:** David W. Jr Polly

**Affiliations:** 1 Department of Orthopaedic Surgery, University of Minnesota, Minnesota, USA

**Keywords:** Patients, Discectomy, Back Pain

Dear Editor,

Farzanegan et al. have presented an important and interesting study looking at the patient reported outcomes using the SF-36 after lumbar discectomy for low back pain (1). This is an important study because it uses patient reported outcomes to evaluate a common problem and a common intervention. In addition the SF-36 can now be used to evaluate any medical intervention and convert that change in to quality adjusted life years (QALYs). The use of QALYs allows comparison of the treatment benefit across different medical conditions. When combined with cost data, a cost per QALY can be determined. Given limited resources and nearly unlimited demands, this concept allows for the rationale allocation of medical resources in order to achieve the greatest benefit. Different societies and different health care systems will have different thresholds to determine what interventions are cost effective for them, but this is clearly the emerging trend.

There are several limitations of this study. The first limitation is that we do not have sufficient detail to really understand the selection process for the patients included versus those excluded from this study. Were they consecutive patients or some sort of convenience sample? The next limitation is understanding what their pre-operative treatment regimen was. Presumably it was usual care for their locale. The next consideration is that the authors have described the procedure as discectomy for low back pain. The most well accepted indication for discectomy is radiculopathy rather than an axial pain pattern. Finally this is simply a cohort treatment study without a comparator group. Cohort studies almost always demonstrate a greater treatment effect size than do randomized controlled trials.

Presuming that the cohort had failed usual therapy, an alternative treatment strategy might include cognitive behavioral therapy as advocated by Fairbanks (BMJ 2005) (2). This demonstrated a significant effect size on what might be a comparable cohort. Similarly Brox and colleagues have demonstrated no incremental benefit of fusion compared to cognitive therapy for patients with chronic low back pain (3-5). So the intriguing aspect of this report is that the patients receiving a lesser intervention than fusion (discectomy) had significant improvement. So we are left with the question of why do these results appear so much better? Is it the patient selection criteria? It appears from the paper that patients had pain for > 3 months. It appears from the SPORT study (Rihn 2011) that duration of symptoms might be a prognostic factor (6). So if the intervention was performed relatively early it may have greater benefit. The challenge to this concept is what is the natural history of these patients and how many would have gotten how much better without the surgery?

Recently the SPORT study has clearly demonstrated the benefit of surgical treatment over usual care for lumbar disc herniation in patients with radiculopathy (Weinstein JAMA 2006). These results have been durable at 4 years (Weinstein Spine 2008) (7, 8). It is difficult to determine if the effect sizes are similar between the Farzanegan study and the SPORT study due to differences in reporting. The SPORT study reported SF36 data using the subscale of bodily pain and physical function. The Farzanegan study reported the SF 36 data using the composite physical component subscale which combines multiple items in a different way. If they would report their data in the same format as the SPORT trial, it would potentially provide some external validation of their results presuming that the Farzanegan study information is available.

Finally and most importantly, the question of importance of the improvement to the patient must be taken into account. Here the concept of minimal clinically important difference (MCID) and substantial clinical benefit (SCB) need to be considered. MCID is the minimum change (improvement) necessary for the patient to be able to tell that they are better (above the signal to noise ratio) (9-11). The SCB is the amount of improvement necessary for the patient to consider the intervention. So MCID is a minimum improvement whereas SCB is the targeted improvement. The MCID for SF 36 PCS is 5.4 points. It appears that these patients did achieve MCID. The authors have reported mean change scores, but it would be important to report the number and percentage of patients achieving MCID rather than just men changes. Similarly for SCB the SF 36 PCS is 6.2.

I would encourage the authors to present their data in the public domain addressing the above points. If they are able to do so, they may open a new chapter on appropriate intervention for low back pain in patients with lumbar disc herniations.
